# Harnessing the Activation of Toll-Like Receptor 2/6 by Self-Assembled Cross-β Fibrils to Design Adjuvanted Nanovaccines

**DOI:** 10.3390/nano10101981

**Published:** 2020-10-07

**Authors:** Soultan Al-Halifa, Ximena Zottig, Margaryta Babych, Mélanie Côté-Cyr, Steve Bourgault, Denis Archambault

**Affiliations:** 1 Department of Chemistry, Université du Québec à Montréal, Montreal, QC H2L 2C4, Canada; soultan.al-halifa@courrier.uqam.ca (S.A.-H.); zottig.ximena_alumine@courrier.uqam.ca (X.Z.); babych.margaryta@courrier.uqam.ca (M.B.); cote-cyr.melanie@courrier.uqam.ca (M.C.-C); 2 The Quebec Network for Research on Protein Function, Engineering and Applications, PROTEO, Quebec, QC G1V 0A6, Canada; 3 The Swine and Poultry Infectious Diseases Research Centre, CRIPA, Saint-Hyacinthe, QC J2S 2M2, Canada; 4 Department of Biological Sciences, Université du Québec à Montréal, Montreal, QC H2L 2C4, Canada

**Keywords:** nanovaccine, fibrils, self-assembly, immune response, immunization, toll-like receptor, TLR2/6, influenza virus, self-assembling peptides, cross-β-sheet

## Abstract

Protein fibrils characterized with a cross-β-sheet quaternary structure have gained interest as nanomaterials in biomedicine, including in the design of subunit vaccines. Recent studies have shown that by conjugating an antigenic determinant to a self-assembling β-peptide, the resulting supramolecular assemblies act as an antigen delivery system that potentiates the epitope-specific immune response. In this study, we used a ten-mer self-assembling sequence (I_10_) derived from an amyloidogenic peptide to biophysically and immunologically characterize a nanofibril-based vaccine against the influenza virus. The highly conserved epitope from the ectodomain of the matrix protein 2 (M2e) was elongated at the N-terminus of I_10_ by solid phase peptide synthesis. The chimeric M2e-I_10_ peptide readily self-assembled into unbranched, long, and twisted fibrils with a diameter between five and eight nm. These cross-β nanoassemblies were cytocompatible and activated the heterodimeric Toll-like receptor (TLR) 2/6. Upon mice subcutaneous immunization, M2e-fibrils triggered a robust anti-M2e specific immune response, which was dependent on self-assembly and did not require the use of an adjuvant. Overall, this study describes the efficacy of cross-β fibrils to activate the TLR 2/6 and to stimulate the epitope-specific immune response, supporting usage of these proteinaceous assemblies as a self-adjuvanted delivery system for antigens.

## 1. Introduction

Over the last decades, vaccination has shifted towards the use of defined molecular components, such as purified microbial antigens. In comparison to conventional vaccines based on live-attenuated and inactivated pathogens, these subunit vaccines are safer formulations, although they tend to be weakly immunogenic and often require the use of immunostimulatory agents, or adjuvants [1]. Recently, usage of nanoparticles, or nanoscale materials, as delivery systems for antigenic components has shown great potential to promote long-lasting immune responses with minimal safety issues. A large diversity of nanoparticles has been evaluated as antigen carriers, including inorganic and polymeric-nanoparticles, polysaccharide-complexes, liposomes, and self-assembled protein nanostructures [2,3]. By tuning their size, supramolecular arrangement, shape, solubility, and surface chemistry, nanoparticles can be designed with tailored physicochemical and immunological properties [4]. One advantage of these nanomaterials resides in their size, with at least one dimension at the nanometer level, closely mimicking most invading pathogens and serving as danger signals for the immune system [5]. These nanovaccines contain the antigen(s) either encapsulated or decorated on the surface, leading to its physical and biological stabilization, its protection from proteolytic degradation, its sustained release, and/or to an increase of its internalization and processing by antigen-presenting cells (APCs) [6]. Interestingly, some nanoparticles have also shown intrinsic immunomodulatory activity associated with stimulation of APCs [7]. Ideally, nanomaterials used as subunit vaccine platforms need to be biocompatible, biodegradable, to show appropriate physicochemical and enhanced biological stability, and to tolerate the functionalization of a diversity of antigens, from oligosaccharides to large proteins [8,9]. Among materials used for subunit nanovaccines, proteins that self-assemble into well-organized repetitive antigen displays have attracted interest for their high biocompatibility, molecular specificity, and multivalency [10]. The best-known protein nanovaccines are virus-like particles (VLPs), composed of viral structural proteins that lack nucleic acid sequences [11], which are currently used as vaccines against the hepatitis B virus and the human papilloma virus [12]. Nonetheless, their complex production, low shelf-stability, presence of biological contaminants, and/or difficult characterization could limit the use of VLPs.

In this context, synthetic peptides have emerged as promising alternative building blocks for the construction of proteinaceous nanostructures for the delivery of antigens. Peptides are biocompatible and efficiently prepared by solid phase synthesis, which facilitates conjugation of biomolecules, purification, and characterization [13,14]. By varying the peptide sequence and/or the conditions of self-assembly, a virtually infinite array of supramolecular architectures with tailored structural, chemical and immunological properties can be prepared. Peptide amphiphilic micelles [15,16,17], synthetic coiled-coil nanoparticles [18,19,20], as well as fibrils and filaments [21,22,23] have been shown to increase the immunogenicity of grafted peptide epitopes. For instance, linear and unbranched fibrils assembled from the cross-β-sheet quaternary motif have been frequently reported to elicit strong humoral immune responses against the conjugated epitopes. Most fibrillar nanovaccines evaluated so far are based on a short self-assembling sequence known as Q_11_ (QQKFQFQFEQQ) [21,22,24]. Fibrils assembled from Q_11_ peptide, with fibril lengths over one µm, have shown to boost the epitope-specific antibody response upon mice immunization against a diversity of grafted T- and/or B-cell epitopes. Recently, we have identified a novel short β self-assembling sequence (I_10_; SNNFGAILSS) for the preparation of a fibrillar nanovaccine, which derived from the amyloidogenic peptide islet amyloid polypeptide (IAPP) [25]. A chimeric peptide comprising the 18-mer E2EP3 epitope from the E2 glycoprotein of the chikungunya virus and the I_10_ peptide led to the formation of linear fibrils with the epitope exposed on their surface. Mice immunized with these fibrils showed a robust IgG response against the E2EP3 epitope, which was dependent on self-assembly and did not require co-injection of an additional adjuvant, demonstrating the potential of this sequence for the preparation of synthetic nanovaccines. Nonetheless, the self-assembling robustness and the intrinsic immunogenecity of the I_10_ delivery nanoplatform have not been validated with other antigens so far. Particularly, whereas hypotheses have been proposed to explain the immunostimulatory properties of cross-β fibrils, such as depot effect, increased phagocytosis and/or activation of APCs, the precise mechanisms of this adjuvant effect remain partially elusive, precluding translation into clinics.

**Figure 1:**
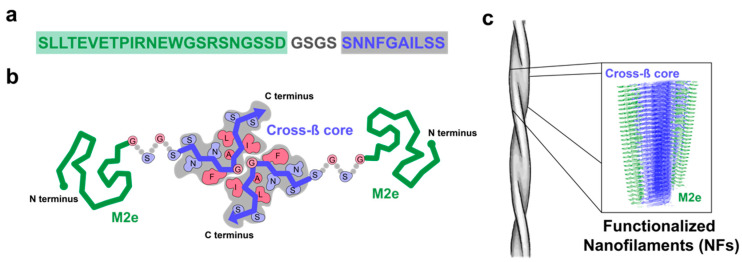
Schematic representation of cross-β fibrils decorated with the 23-mer negatively charged epitope (M2e) epitope. (**a**) Sequence of the M2e-I_10_ peptide comprising the epitope derived from the N-terminal domain of M2 protein of the influenza virus (green), a short flexible tetrapeptide linker (gray), and the self-assembling peptide I_10_ (in blue). Please note that the Cys residues at positions 17 and 19 were substituted to Ser to avoid undesired oxidation and/or formation of aberrant disulfide bonds, without affecting immunogenicity of M2e and affinity/selectivity of the resulting antibodies [29]. (**b**) Schema of two M2e-I_10_ peptides interacting via a cross-β motif. (**c**) Representation of fibrils and arrangement of the cross-β supramolecular structure with the M2e epitope exposed on the surface.

In this study, we evaluated the self-assembling capacity and the adjuvanticity of the I_10_ peptide sequence by using an immunogenic epitope derived from the ectodomain of the matrix protein 2 of the influenza A virus (Figure 1). This M2e linear peptide epitope (SLLTEVETPIRNEWGCRCNDSSD) is highly conserved among different influenza A strains and therefore, represents a potential candidate for a universal influenza vaccine [26,27,28]. Our results showed that the self-assembly of M2e-I_10_ peptide into linear cross-β twisted fibrils tolerates the N-terminal conjugation of M2e, leading to the exposition of the epitope on the surface. This fibrillar nanoplatform raised a robust M2e-specific immune response in mice, likely involving engagement of innate immunity through activation of the heterodimeric Toll-like receptor (TLR) 2/6. 

## 2. Materials and Methods 

### 2.1. Peptide Synthesis, Purification, and Characterization 

Peptides were synthesized by solid phase peptide synthesis (SPPS) using a Fmoc/tBu strategy and 2-(6-chloro-1-H-benzotriazole-1-yl)-1,1,3,3-tetramethylaminium hexafluorophosphate (HCTU) as a coupling agent, as previously described [30]. Crude peptides were purified by preparative HPLC using a C_18_ column and a linear gradient of acetonitrile in H_2_O/TFA (at 0.6% v/v). Collected fractions were analyzed by analytical HPLC (C_18_) (Agilent Technologies, Santa Clara, CA, USA) and ‘time of flight’ mass spectrometry (LC/ESI-TOF)((Agilent Technologies, Santa Clara, CA, USA). Fractions corresponding to the desired peptide with a purity of over 95% were pooled and lyophilized.

### 2.2. Peptide Self-Assembly

Lyophilized peptide was solubilized at 500 µM, unless otherwise specified, in 50 mM Tris-HCl, pH 7.4 and sonicated for 5 min. Self-assembly was performed at room temperature (RT) under constant rotary agitation at 40 rpm for up to 168 h. At different times of self-assembly, aliquots were taken and the assemblies were characterized by a combination of biophysical approaches, as described below.

### 2.3. Critical Aggregation Concentration

Stock solution of pyrene (1 mM) was prepared in ethanol, diluted in 50 mM Tris-HCl buffer, pH 7.4, and sonicated until complete dissolution. Pyrene was employed at a final concentration of 2 µM in the peptide solution of the M2e-I_10_ peptide, reaching 0.2% ethanol. This ethanol concentration was considered to have no effect on the aggregation behavior of the M2e-I_10_. The excitation wavelength was set at 335 nm and the emission spectra were recorded from 350 nm to 450 nm. CAC was determined by plotting the ratio of fluorescence intensity (λ_373 nm_/λ_384 nm_) as function of the concentration. Intersection of the two linear fits was used to determine the CAC. This experiment was performed in triplicate and representative data are presented. The CAC was determined by averaging the data from three separate experiments.

### 2.4. Absorbancce and Fluorescence Spectroscopy

Kinetics of self-assembly at 500 µM in Tris-HCl (50 mM, pH 7.4) was assessed by absorbance measurements at 400 and 600 nm. Thioflavin T (ThT) fluorescence endpoint was used to monitor formation of cross-β quaternary structure. Assemblies were diluted in Tris-HCl (50 mM, pH 7.4) to reach 100 μM and ThT was added to a final concentration of 40 μM. Fluorescence was measured with excitation wavelength at 440 nm and emission spectra were recorded from 450 to 550 nm. Data of at least three experiments were averaged and expressed as the mean ± S.D.

### 2.5. Circular Dichroism Spectroscopy

At the desired time of self-assembly, peptide solutions were diluted in Tris-HCl (50 mM, pH 7.4) to reach a concentration of 100 μM and incorporated into a 1-mm path-length quartz cell. Far-UV circular dichroism (CD) spectra (Jasco Inc., Easton, MD, USA) were recorded from 190 to 260 nm. The wavelength step was set at 0.5 nm with an average time of 10 s per scan at each wavelength step. Each collected spectrum was background subtracted with the buffer control. Raw data were converted to mean residue ellipticity (MRE), as previously reported [31]. CD experiments were performed in triplicate and representative data are presented. 

### 2.6. Powder X-ray Diffraction 

Peptide solutions were deposited on an X-ray diffraction lamella and dried overnight. Powder X-ray diffraction (PXRD) was performed using a Bruker D8 Advance X-ray diffractometer (Bruker, Billerica, MA, USA). The current and the voltage were 40 mA and 40 mV, respectively, with a step size of 0.112° s^−1^ in the 2θ range of 5–50°. Diffractograms were analyzed using X’pert data software. Interplanar distances were determined from powder raw pattern (2θ), satisfying Bragg’s condition [32].

### 2.7. Atomic Force Microscopy 

Peptide solutions were diluted to 50 μM in 1% acetic acid and immediately applied to freshly cleaved mica. Micas were washed with deionized water and were air-dried for 24 h. Images were acquired on a Veeco/Bruker Multimode AFM using the Scan-Assyst in air mode with a silicon tip (2–12 nm tip radius, 0.4 N m^−1^ force constant) on a nitride lever. Images were obtained at 0.9 or 0.5 Hz and 1024 scan/min and analyzed using the Gwyddion software. The length of at least 150 individual fibrils per image were manually measured and length distribution was plotted using a frequency distribution. Non-linear fitting was obtained using a Gaussian distribution. For height analysis, the mask grain by threshold function was applied to discriminate the background form the fibrils. The heights of over 500 individual fibrils per image were plotted using the frequency distribution and non-linear fitting was obtained using a Gaussian distribution. 

### 2.8. Transmission Electron Microscopy

Peptide solutions were diluted to 50 μM and applied to glow-discharged copper carbon-coated 400-mesh grids. Samples were negatively stained with 1.5% (w/v) uranyl formate for 1 min and air-dried for 24 h. For immunogold staining, grids with adsorbed fibrils were floated on droplets of 1% bovine serum albumin (BSA) to block the non-specific binding. After incubation with the M2e-specific primary antibody (1:1000) for one hour, grids were washed with 1% BSA in PBS, floated on droplets of gold-conjugated goat anti-rabbit IgG (1:500) for 1 h, washed with distilled water, and stained with 1.5% (w/v) uranyl formate. Grids were examined using a FEI Tecnai 12 BioTwin microscope operating at 120 kV and equipped with an AMT XR80C CCD camera system (Thermo Fisher Scientific, Waltham, MA, USA)).

### 2.9. Enzyme-Linked Immunosorbent Assay for Epitope Accessibility 

High-binding ELISA plates were coated with 2 μM of peptide (M2e, NFs, M2e-I_10_, and M2e-NFs) overnight at 4 °C. Wells were washed and blocked with PBS 0.05% Tween-20 (PBS-T) containing 1% (w/v) BSA for 1 h. The coated plates were treated with an anti-influenza A M2 monoclonal primary antibody (14C2) (1:1000) for 3 h at room temperature (RT). Plates were washed three times and incubated with peroxidase-conjugated rabbit anti-mousse IgG (1:10,000). The peroxidase signal was detected using 3,3′-5,5′-tetramethyl benzidine (TMB) and by measuring the absorbance at 450 nm after stopping the reaction with sulfuric acid.

### 2.10. Measurement of Zeta potential

Measurements were carried out at a concentration of peptides of 500 µM in 50 mM Tris-HCl pH 7.4, using a ZetaPlus instrument (Malvern Panalytical, St-Laurent, QC, Canada). operated at room temperature. Each measurement corresponds to a triplicate of 10 runs per analysis.

### 2.11. Cell Viability Assays 

J774.1 cells were grown in Dulbecco’s modified Eagle medium (DMEM) supplemented with 10% (v/v) fetal bovine serum (FBS), 50 U/mL penicillin, and 50 μg/mL streptomycin at 37 °C under a humidified atmosphere of 5% CO_2_. DC2.4 cells were grown in RPMI-1640, supplemented with 10% FBS, 2 mM L-Glutamine, 0.1 mM non-essential amino acids, 25 mM HEPES buffer solution, and 0.05 mM β-Mercaptoethanol. For live/dead viability assays, cells were cultured in a 24-well plate at a density of 250,000 and 180,000 cells/well for J774.1 and DC2.4, respectively. For metabolic assays, cells were seeded in black-wall, clear-bottom 96-well plates at a density of 25,000 and 18,000 cells/well, respectively. After overnight incubation, cells were treated, for 16 h with M2e-NFs or IAPP peptide at final concentrations of 150 µM and 50 µM, respectively, or with the Tris-HCl buffer vehicle control. The medium was removed and live/dead reagent solution (4 μM ethidium homodimer-1; 2 μM calcein-AM) in sterile PBS solution (pH 7.4) was added in each well. After incubation for 45 min at room temperature, plates were imaged using a fluorescent microscope. At least three images per well were taken and processed using ImageJ software. The percentage of cell viability was calculated as the viable cells (green labeled) over the total number of cells (green + red). Metabolic activity of treated cells was also evaluated by the resazurin reduction assay. After peptide treatment, cells were incubated with 50 µM of resazurin for 3 h and the fluorescence intensity was measured with an excitation at 560 nm and an emission at 590 nm. Cell viability (in %) was calculated from the ratio of the fluorescence of the treated cells to the buffer-treated cells. Data of at least four experiments were averaged and expressed as the mean ± S.D.

### 2.12. Evaluation of TLR2/6 Activation

HEK-Blue™ hTLR2-TLR6 cells (InvivoGen, San Diego, CA, USA) were cultivated in DMEM supplemented with 4.5 g/L glucose, 10% (v/v) fetal bovine serum, 100 U/mL penicillin, 100 μg/mL streptomycin, 100 μg/mL normocin, 2 mM L-glutamine, and 100 μg/mL HEK-Blue selection. At 50–80% confluency, cells were seeded in HEK-Blue detection medium (InvivoGen, San Diego, CA, USA) at a density of 50,000 cells/well in a 96-well plate containing, or not, the peptide assemblies at the indicated concentrations. Pam2CSK4 was used as positive control at a concentration of 10 ng/mL. After 16 h incubation at 37 °C in 5% CO_2_, absorbance was monitored at 630 nm. 

### 2.13. Mice Immunization 

Protocols were approved by the institutional committee (CIPA: Institutional Animal Care and Use Committee of the *Université du Québec à Montréal*) according to the regulation of the Canadian Council for Animal Care. Peptides were dissolved in endotoxin-free sterile Tris-HCl (50 mM, pH 7.4) at 2 mM and self-assembled as described above. Stock solutions were then diluted to reach a concentration of 0.5 mM in endotoxin-free sterile PBS. Six-week-old female BALB/c mice (8 mice per group) were immunized subcutaneously with 100 μL (50 nmol/mice) of synthetic peptide (M2e), M2e-NFs with, or without, aluminum hydroxide gel (Alhydrogel adjuvant (Alum) 2%). Alum-adjuvanted groups received the same volume and peptide dose, prepared by diluting the peptide solution in alum at a 1:1 volume ratio. Mice received two boosts at days 15 and 29 post-primary immunization with 100 μL, each containing 50 nmol of peptides. Control mice were immunized using the same volume of PBS. Blood samples were collected from the saphenous vein at days 0-, 14-, and 28-post primary-immunization. Mice were sacrificed two weeks after the final boost (day 42) and sera were collected from cardiac puncture.

### 2.14. Determination of Anti-M2e Antibody Titers

Antibody titers were determined using an indirect ELISA assay. Plates were coated overnight at 4 °C with 2 μg/mL of M2e peptide diluted in sodium carbonate 0.05 M (pH 9.6). After extensive washing with PBS-T, plates were blocked with 1% (w/v) bovine serum albumin (BSA) solution for 1 h. Determination of whole IgG was performed using 100 μL of a serial dilution of mouse sera (starting point at 1 : 65) in PBS-T (1% BSA), while isotype IgG titers were obtained by a dilution of 1:1600 of antisera (IgG2a, IgG2b, IgG3) or 1:12800 (IgG1). After 3 h incubation and extensive washes, HRP-conjugated goat anti-mouse whole IgG (H+L) (G-21040) (1:10,000), IgG1 (A10551) (1:10,000), IgG2a (A-10685) (1:5000), IgG2b (M32407) (1:5000), and IgA (62-6720) (1:10,000) were added for 1 h. Plates were washed and the HRP signal was detected using TMB substrate. To determine antibody titers, the optical density (450 nm) was compared to the cutoff value. The endpoint antibody titers were calculated by regression analysis, plotting serum dilution versus the absorbance with the following regression curve equation: y = (b + cx)/ (1 + ax). Endpoint titers were defined as the highest dilution resulting in an absorbance value twice that of blank points (points without immune serum) [33]. 

### 2.15. Data Analysis

For animal experiments, data were expressed as arithmetic means ± standard errors of the means (SEM). One-way analysis of variance (ANOVA) with multiple comparisons was used (GraphPad software, San Diego, CA, USA). *p* values of < 0.05 were considered significant; levels of significance are indicated on the graphs by asterisks: *, *p* = 0.01; **, *p*= 0.001; ***, *p* = 0.0001; and ****, *p* < 0.0001.

## 3. Results and Discussion

### 3.1. M2e-I_10_ Self-Assembles into Twisted Fibrils with a Cross-β-Sheet Quaternary Architecture

**Figure 2:**
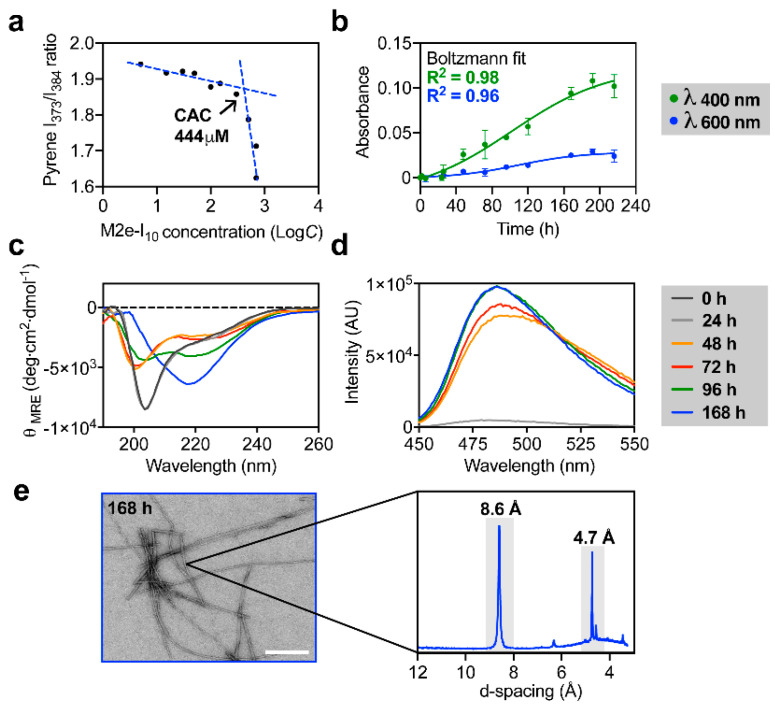
Biophysical characterization of the self-assembly of M2e-I_10_ into fibrils. (**a**) Determination of critical aggregation concentration (CAC) by plotting the pyrene I_373_/I_384_ fluorescence ratio *vs.* M2e-I_10_ concentration. (**b**) Absorbance measurement over time of self-assembly of M2e-I_10_. Raw data and non-linear Boltzmann fit are presented. (**c**,**d**) Kinetics of self-assembly of M2e-I_10_ measured by far-UV circular dichroism (CD) spectroscopy and (**d**) ThT fluorescence, with excitation at 440 nm. (**e**) Representative TEM image of M2e-I_10_ assembled for 168 h (left panel) and diffraction pattern obtained by powder X-ray diffraction (PXRD) (right panel).

M2e-I_10_ chimeric peptide (Figure 1) was prepared by solid phase synthesis and stored at −20 °C under lyophilized form to avoid uncontrolled and premature self-assembly [25]. The ability of the β peptide I_10_ to self-assemble into supramolecular structure upon N-terminal conjugation of the 23-mer M2e peptide epitope was initially evaluated by determining the critical aggregation concentration (CAC) using pyrene fluorescence (Figure 2a). Pyrene is a small fluorogenic dye whose entrapment within hydrophobic core leads to a change of its optical properties [34]. The results showed that M2e-I_10_ aggregates with a CAC value of 444 µM, indicating that a working concentration above this CAC is sufficient to promote self-assembly. Next, the kinetics of self-assembly was evaluated by monitoring biophysical parameters overtime. M2e-I_10_ peptide was incubated at 500 µM, i.e., above the CAC, under constant rotary agitation at room temperature and the solution was periodically analyzed by turbidity, circular dichroism (CD) spectroscopy, and thioflavin T (ThT) fluorescence. The growth of amyloid-like fibrils in suspension is known to be associated with an increase of solution turbidity [35,36]. Accordingly, by measuring the absorbance of the solution at 400 and 600 nm over incubation time, we observed that a plateau was attained after 160 h, suggesting that under these conditions, self-assembly reaches equilibrium (Figure 2b). Boltzmann fittings with R-squared of 0.98 and 0.96 for absorbance measurements at 400 and 600 nm, respectively, further confirmed that the plateau was reached. CD spectroscopy revealed a secondary conformational transition from a mixture of random coil and α-helix (two minima at 200 and 222 nm) to a β-sheet (one minimum at 217 nm) occurring between 96 to 168 h incubation (Figure 2c), in agreement with turbidity measurement and what has been reported for amyloid-related assemblies. ThT fluorescence, which reports the formation of cross-β-sheet quaternary structure [37,38], revealed that M2e-I_10_ peptide assembled into ThT-positive structure after 48 h, and that a plateau of ThT fluorescence was reached between 96 and 168 h. 

Transmission electron microscopy (TEM) analysis of the peptide incubated under constant rotary agitation for 168 h showed the formation of long, linear, and unbranched fibrils (Figure 2e, left panel). Finally, the cross-β quaternary supramolecular structure was assessed by X-ray diffraction (XRD). Powder XRD measurements revealed a diffraction pattern characterized with two sharp peaks (Figure 2e, right panel). Those peaks, also known as Bragg reflections, were at 4.7 and 8.6 Å periodic spacing. The 4.7 Å meridional reflection corresponds to the space between hydrogen-bonded β-strands, a typical signature of the cross-β-sheet structure, whereas the 8.7 Å spacing corresponds to inter-sheet distance [32,39]. 

**Figure 3:**
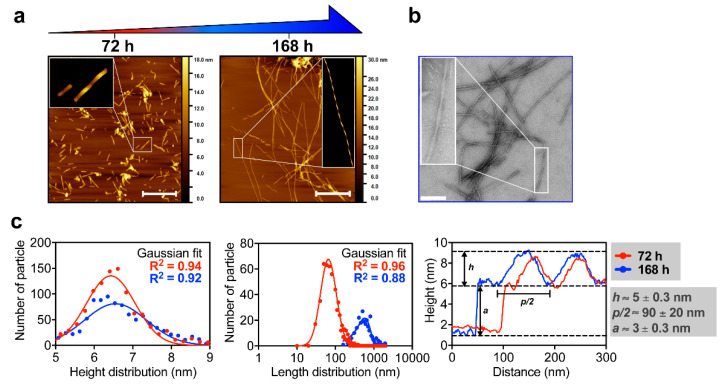
Morphological characterization of M2e-NFs. (**a**) Representative atomic force microscopy (AFM) images of M2e-NFs taken after 72 h and 168 h of self-assembly showing the morphology of the nanostructures. Scale bar is 200 nm. (**b**) Representative TEM image of M2e-NFs obtained after 168 h self-assembly. Scale bar is 100 nm. (**a**,**b**) Insets are zoomed in region of the images taken at high resolution with further digital treatments to highlight the twisted morphology of NFs. (**c**) Height and length distribution, and height profiles of M2e-NFs at 72 h (red line) and 168 h (blue line) self-assembly extracted from AFM images. *h*, *p/2* and *a* on the height extraction profile graph correspond to height, half of the pitch, and amplitude, respectively.

Atomic force microscopy (AFM) analysis of M2e-I_10_ assembled for 72 h showed the presence of a heterogeneous population of short fibrils (Figure 3a). As observed by AFM and TEM imaging, these short fibrils evolved into long and mature nanofilaments (NFs) upon aging, i.e., after 168 h incubation (Figure 3b). The Gaussian length distribution of these assemblies showed an increase in the average length from 97.2 ± 53.4 nm to 782.4 ± 508.6 nm for M2e-I_10_ peptide assembled for 72 h and 168 h, respectively (Figure 3c). In sharp contrast, the Gaussian height distribution of fibrils remained constant overtime, i.e., (*h*) = 6.4 ± 0.2 nm after 72 h and (*h*) = 6.6 ± 0.8 nm after 168 h, indicating that elongation of short filament into long and linear fibrils does not impact their diameter. This suggests that elongation from short fibrils to extended NFs likely occurs on one dimension along the fibril axis. Extraction of height profile combined with zoomed AFM images of the NFs (insets in Figure 3a,b) showed that NFs (after 72 and 168 h of assembly) are twisted and have a ribbon-like mesoscopic morphology, with an absolute height (*h*) of about 5 ± 0.2 nm, a half-pitch (*p/2*) of about 90 ± 20 nm, and an amplitude (*a*) of 3 ± 0.2 nm (Figure 3c). The absolute height (*h*) correlated closely with the height distribution measurement, which includes the amplitude of the twist and gives an average measure of the height. The close similarity between the extraction profiles suggests that the symmetry is conserved during fibril growth and supports the one-dimension elongation hypothesis aforementioned. Taken together, biophysical analyses revealed that the functionalization of the self-assembling I_10_ peptide with the M2e epitope does not hinder its self-assembly, leading to twisted fibrils with a prototypical cross-β quaternary structure. These observations indicate that the self-assembling propensity of I_10_ is sufficiently robust to tolerate the N-terminal functionalization of different antigenic determinants, such as M2e and the E2EP3 immunogenic epitope from the chikungunya virus [25]. 

### 3.2. The M2e Epitope is Accesible on the Surface of the Fibrils

**Figure 4:**
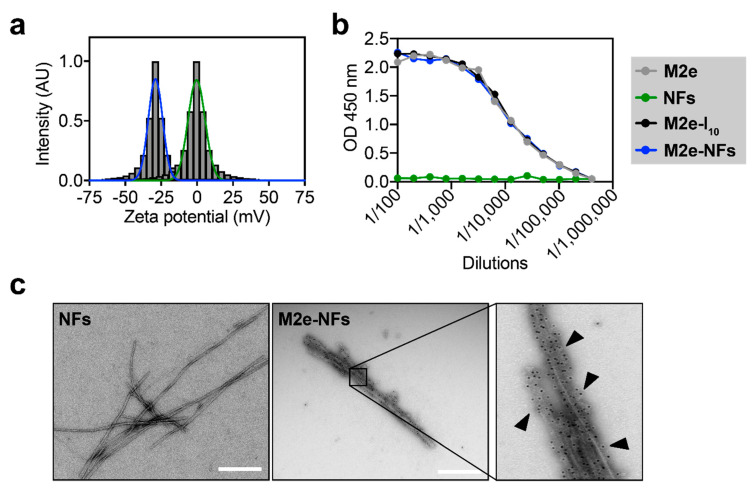
M2e-NFs display high density of the M2e epitope at the surface. (**a**) Measurement of zeta potential indicating the surface charge of M2e-NFs and of non-functionalized fibrils (NFs). (**b**) ELISA analysis of M2e peptide, NFs, monomeric M2e-I_10_, and M2e-NFs. (**c**) TEM images of NFs and M2e-NFs after immunogold staining with anti-M2 primary antibody and gold-conjugated secondary antibody.

We evaluated if the M2e epitope is exposed on the fibril surface and not buried inside the hydrophobic core of the nanostructures, which could allow direct recognition by B cells [10,40,41]. First, measurement of zeta potential was assessed to evaluate the electrostatic charge on the surface of the M2e-NFs in comparison to naked I_10_ fibrils. In fact, whereas the self-assembling platform I_10_ displays a net charge of +1 at pH 7.4, the M2e peptide has a net charge of −2 at the same physiologic pH (Figure 1a). Zeta potential measurements of the non-functionalized fibrils (NFs) and M2e-NFs were −0.31 ± 5.4 mV and −28.97 ± 4.5 mV, respectively, suggesting that the negatively charged M2e sequence is exposed on the fibril surface (Figure 4a). Next, ELISA using M2e-specifc antibody was performed to evaluate the accessibility of the epitope on the fibrils. As observed in Figure 4b, the M2e epitope on the monomeric M2e-I_10_ peptide and the assembled M2e-NFs was accessible to a comparable extent to the M2e peptide alone. Absence of antibody binding to the naked NFs confirmed the specificity of the assay. Finally, availability of the M2e epitope on the surface of the M2e-NFs was confirmed by TEM immunogold-labelling using a secondary antibody labelled with a 10-nm gold nanoparticle. TEM images clearly showed the homogenous distribution of the gold nanoparticles along the M2e-NFs in contrast to naked NFs, confirming the specificity of the immuno-assay (Figure 4c). Overall, zeta potential and immuno-labelling showed that NFs display a high density of repetitive epitope on the surface, mimicking the ordered native conformation of virus surface. 

### 3.3. M2e-NFs are Cytocompatible

**Figure 5:**
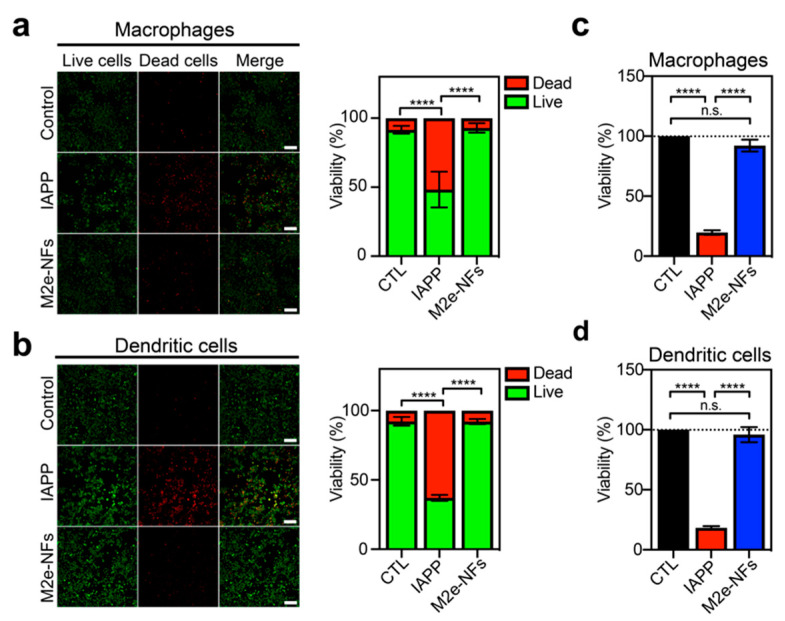
M2e-NFs are fully cytocompatible. (**a**,**b**) Qualitative and quantitative live/dead assay of M2e-NFs and amyloidogenic peptide islet amyloid polypeptide (IAPP) showing cell viability of macrophages (**a**) (J774A) and (**b**) dendritic cells (D.C2.4). Left panels are images taken by a fluorescent confocal microscope in which live cells are labelled in green (intracellular esterase activity) and dead cells are red (loss of plasma membrane integrity). Scale bar: 20 μm. (**c**,**d**) Cell viability evaluated from metabolic activity of (**a**) J774A and (**b**) D.C2.4 treated with M2e-NFs and IAPP. (**a**–**d**) Data represent the mean ± S.D. of at least three experiments performed in triplicate. Results were analyzed using the student’s t-test and statistical difference (between control cells and treated cells) was established at (****) < 0.0001.

While cross-β proteinaceous assemblies have shown potential for applications in biomedicine and as nanomaterials [42], this quaternary structural motif is closely associated with amyloid fibrils, whose tissue deposition and accumulation have been historically associated with numerous diseases [43], raising some concerns regarding their usage in the biomedical field. However, recent compelling biophysical and biochemical evidence [43,44,45] as well as the identification of a diversity of functional amyloids in numerous species [46] have suggested that mature amyloid fibrils are poorly toxic and that the toxicity is mainly ascribed to transient oligomers and/or pre-fibrillar proteospecies. Nonetheless, we evaluated the cytocompatibility of M2e-NFs using macrophages (J774.1) and dendritic cells (DC2.4). Cells were incubated overnight in the presence, or absence, of 150 μM M2e-NFs (determined relatively to the monomer), or with 50 μM monomerized IAPP, used as a positive control of amyloid-related cytotoxicity [47]; and cell viability was evaluated using the live/dead assay and by measuring metabolic activity. Fluorescence microscopy images show a similar green-to-red ratio for cells treated with M2e-NFs and with the PBS control for macrophages (Figure 5a) and dendritic cells (Figure 5b). The green fluorescence is associated with intracellular esterase activity of metabolically active cells, whereas the red fluorescence reports cells that lose the integrity of their plasma membrane. By measuring the green-to-red fluorescence ratio, the cell viability was 91.5 ± 2.8% for macrophages and 92.3 ± 3.0% for dendritic cells treated with M2e-NFs. In sharp contrast, cells treated with IAPP showed an important increase of red cells; corresponding to a cell viability of 48.3 ± 13.0% for macrophages and 37.1 ± 2.1% for dendritic cells. Measurement of the reduction of resazurin to resorufin, an indicator of metabolically active cells, confirmed the cytocompatibility of the M2e-NFs observed by the live/dead assay (Figure 5c,d). 

### 3.4. M2e-NFs Activate the Heterodimeric Toll-Like Receptor 2/6 

**Figure 6:**
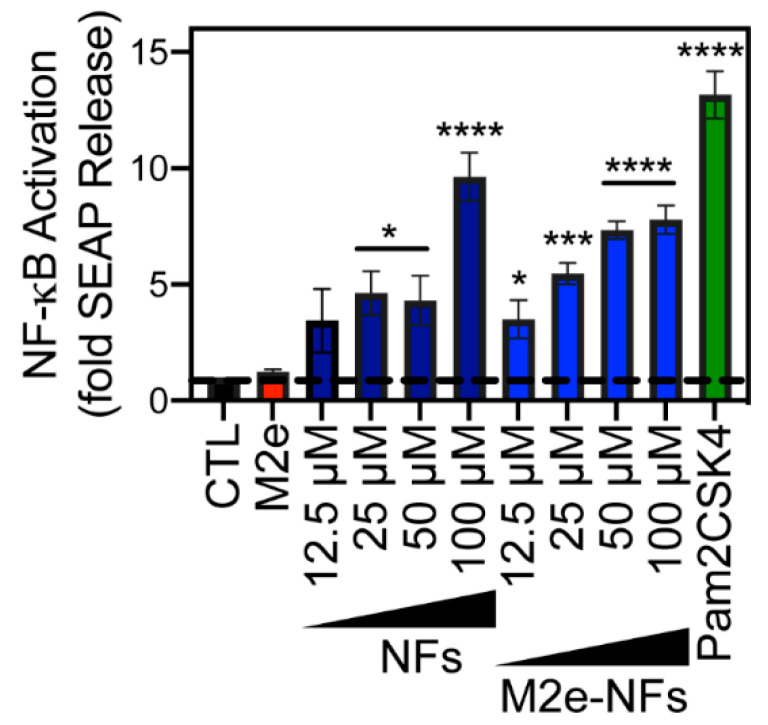
M2e-NFs activate TLR2/6. HEK-Blue hTLR2-TLR6 cells were stimulated with increasing concentrations of NFs and M2e-NFs (ranging from 12.5 to 100 µM), 150 µM of M2e peptide, 10 ng/mL of Pam2CSK4 or with the PBS buffer (CTL) for 16 h. NF-κB-induced SEAP activity was quantified using HEK-Blue detection media. Data represent the mean ± S.D. of at least three experiments performed in triplicate. Results were analyzed using the student’s t-test and statistical difference (between control cells and treated cells) was established at (*) 0.01; (**) 0.001; (***) 0.0001; (****) < 0.0001.

The immunostimulatory properties of cross-β fibrils and their capacity to enhance the specific immune response against different grafted antigens have been reported several times over the last decade [21,24,25,48,49]. Nonetheless, the precise molecular basis of this effect remains unknown. While formation of a depot at the injection site and protection of the antigen from proteolytic digestion are potential mechanisms of this adjuvant effect, studies have revealed that the cross-β-sheet supramolecular structure of fibrils can directly activate the innate immune responses. For instance, cross-β fibrillar aggregates assembled from amyloid β-peptide and IAPP have been shown to activate the heterodimeric TLR2/6 [50,51]. TLRs are important pattern recognition receptors (PRRs) of the host APCs, which are known to potentiate the epitope-specific response by bridging innate and adaptive immunity [52]. Activation of the membrane heterodimeric TLR2/6 triggers the NF-kB pathway, leading to the stimulation and maturation of APCs and secretion of cytokines [53]. According to these previous studies, we evaluated the capacity of NFs and M2e-NFs to engage the TLR2/6 by using HEK-Blue hTLR2/6 cells, which overexpress this PRR in addition to a NF-kB-inducible reporter gene SEAP (secreted embryonic alkaline phosphatase). Incubation of these cells with M2e-NFs, with concentrations ranging between 12.5 to 100 µM, led to a concentration-dependent activation of NF-kB (Figure 6). This effect was not associated with the M2e epitope, as 150 μM soluble M2e peptide did not increase the release of SEAP in the culture media, whereas naked NFs, i.e., without the M2e epitope, showed comparable engagement of TLR2/6 to the M2e-NFs (Figure 6). Although the activation of the NF-kB pathway by the TLR2/6 agonist Pam2CSK4 was significantly higher, 50 μM of M2e-NFs led to an 8-fold increase of SEAP activity in comparison to the M2e and PBS-treated control cells. To the best of our knowledge, this is the first report of the capacity of self-assembled cross-β fibrillar nanovaccines to activate TLR2/6, which likely contributes to their immunostimulating properties. Besides, TLR engagement resulting in the activation of the transcription factor NF-kB, acts as a first priming signal of the inflammasome by the upregulation of NLRP3 [54,55]. Thus, considering that some studies have shown that cross-β fibrils derived from the amyloid β-peptide [56] and IAPP [57,58] can activate the NLRP3 inflammasome, the stimulation of TLR2/6 by M2e-NFs could also result on inflammasome priming, a hypothesis that will need to be further investigated.

### 3.5. Cross-β Fibrils Potentiate the Anti-M2e Specific Immune Response 

**Figure 7:**
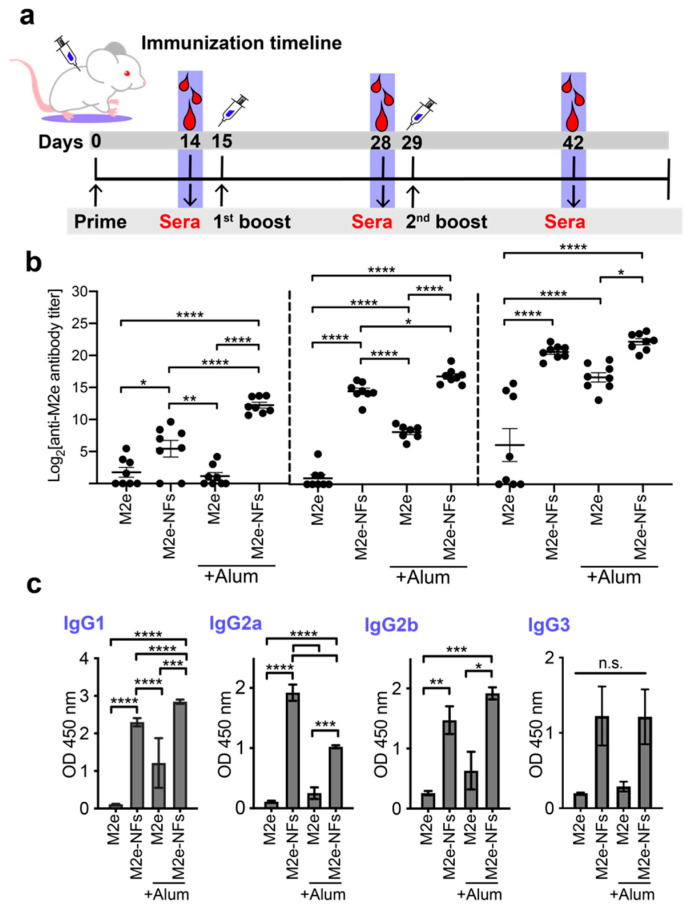
Antibody specific immune response in subcutaneously immunized mice. (**a**) Schematic representation of the immunization schedule. (**b**) M2e-specific serum IgG antibody titers of mice immunized with M2e peptide, M2e + Alum, M2e-NFs, and M2e-NFs + Alum, 50 nmol/dose. (**c**) IgG subtypes from sera collected at day 42 PPI. Data are expressed as arithmetic means ± S.E.M. One-way analysis of variance (ANOVA) with multiple comparisons was used and P values of < 0.05 were considered significant with levels of significance indicated on the graphs by asterisks: *, *p* = 0.01; **, *p* = 0.001; ***, *p* = 0.0001; and ****, *p* < 0.0001.

The immunostimulating properties of M2e-NFs were evaluated by immunizing six-week-old female BALB/c mice and following the M2e-specific antibody response overtime. Mice were subcutaneously (SC) immunized three times at 14-day intervals with 50 nmole/dose of M2e-NFs or M2e peptide, with and without aluminum salts (Alum), as presented in Figure 7a. At days 14-, 28-, and 42-post-primary immunization (PPI), sera were collected and the M2e-specific total IgG titer was determined by ELISA. After the primary immunization, i.e., at day 14 PPI, mice immunized with the M2e-NFs +/− Alum showed a significant increase of anti-M2e specific IgG levels, albeit the antibody titer was higher for the formulation supplemented with Alum (Figure 7b). In sharp contrast, sera of mice immunized with the M2e peptide (+/−Alum) revealed a very low anti-M2e immune response after a single immunization. Fourteen days after the first boost, the M2e-NFs +/− Alum formulations led to a robust anti-M2e immune response. Particularly, the anti-M2e IgG titer at day 28 was significantly higher for the M2e-NFs compared to the M2e + Alum formulation supporting the self-adjuvanticity of the I_10_ nanoplatform. Sera collected at day 42 PPI, i.e., after two immunization boosts, revealed similar anti-M2e IgG titers for the M2e-NFs and the M2e-NFs + Alum groups. This indicates that M2e-NFs alone trigger a somewhat mild immune response after a single immunization, which increases after the 1^st^ and the 2^nd^ boosts to eventually reach the IgG levels observed with mice immunized with M2e-NFs + Alum. 

The IgG subtypes from the final intra-cardiac bleed at day 42 PPI were determined in order to gain an indication of the polarization of immune responses, as the ratio between IgG isotypes is a good indicator of the Th1/Th2 balance [59]. As expected, the M2e-NFs administered with or without Alum stimulated the robust production of IgG1, IgG2a, and IgG2b in comparison to the M2e +/− Alum groups (Figure 7c), further highlighting the contribution of the fibrillar nanoplatform on immunogenicity. When comparing M2e-NFs with M2e-NFs + Alum, both formulations stimulated the production of similar levels of IgG2b and IgG3, but different levels of IgG1 and IgG2a. IgG1 antibodies are generally associated with a Th2-type response while the IgG2b is commonly associated with a polarized Th1-type immune response [60,61]. The addition of Alum in the M2e-NFs vaccine resulted in higher level of IgG1, in agreement with the known capacity of Alum to polarize the immune response towards Th2 [53,62]. Interestingly, M2e-NFs administered in absence of Alum induced a balanced Th1/Th2 immune response, which is suitable to fight against numerous viral infection [4]. Results of mice immunization further emphasizes the self-adjuvant effect of fibrils assembled from the I_10_ peptide, as previously reported with the E2EP3 epitope derived from the chikungunya virus [25]. In fact, cross-β-sheet fibrils respectively assembled from I_10_ and Q_11_ peptides have shown comparable immunogenicity to other protein-based nanostructures, such as VLPs based on the papaya mosaic virus [63] and nanorings assembled from nucleoprotein of respiratory syncytial virus [33]. Nonetheless, comparative studies will need to be performed to compare directly the immunostimulatory properties of peptide-based amyloid fibrils with other proteinaceous nanoscaffolds.

## 4. Conclusions

The results of the present study confirmed the versatility and the robustness of the I_10_ self-assembling sequence as an immunogenic scaffold for subunit vaccines. The self-assembly of I_10_ peptide into long and twisted fibrils tolerated the addition of a 23-mer negatively charged epitope (M2e), as well as an 18-mer positively charged epitope, as previously shown for the E2EP3 epitope derived from the chikungunya virus [25]. Particularly, I_10_-based cross-β assemblies can engage the innate immunity heterodimeric receptor TLR2/6, likely contributing to the immunostimulatory effects observed for antigen delivery platforms based on cross-β-sheet fibrils, as shown herein. As previously reported [25], the conjugation of antigenic determinant to fibrillar nanostructures promotes its internalization by APCs, which constitutes a prerequisite for the initiation of the epitope-specific immune response. Moreover, protection of the antigens from proteolytic degradation and depot effect at the immunization site are also likely contributing to the immunostimulatory properties of cross-β fibrils [10,64]. Besides, it remains critical to evaluate if the immunogenicity of amyloid-like fibrils constitutes an intrinsic characteristic of the cross-β quaternary organization, or if different amyloidogenic sequences can lead to divergent immunogenic potency. Overall, this study emphasized that harnessing the cross-β supramolecular architecture represents a promising strategy to design self-adjuvanted nanoparticles. Nonetheless, limitations regarding cross-β-sheet fibrils, including their high polymorphism in terms of size and shape, their potential accumulation in the host organism because of their resistance to proteolysis, their length in the microscale that could prevent direct draining to the lymph nodes, and potential (cross)seeding of endogenous proteins leading to their aggregation similar to a prion-like effect, will need to be addressed before translating this vaccine technology into clinics. 

## References

[1] Aguilar J., Rodríguez E. (2007). Vaccine adjuvants revisited. Vaccine.

[2] Zaman M., Good M.F., Toth I. (2013). Nanovaccines and their mode of action. Methods.

[3] Boraschi D., Italiani P. (2015). From Antigen Delivery System to Adjuvanticy: The Board Application of Nanoparticles in Vaccinology. Vaccines.

[4] Al-Halifa S., Gauthier L., Arpin D., Bourgault S., Archambault D. (2019). Nanoparticle-Based Vaccines Against Respiratory Viruses. Front. Immunol..

[5] Laval J.-M., Thomas D., Mazeran P.-E. (2000). Nanobiotechnology and its role in the development of new analytical devices. Analyst.

[6] Luo M., Samandi L., Wang Y., Chen Z.J., Gao J. (2017). Synthetic nanovaccines for immunotherapy. J. Control. Release.

[7] Mamo T., Poland G.A. (2012). Nanovaccinology: The next generation of vaccines meets 21st century materials science and engineering. Vaccine.

[8] Kubackova J., Zbytovska J., Holas O. (2020). Nanomaterials for direct and indirect immunomodulation: A review of applications. Eur. J. Pharm. Sci..

[9] Yan X., Zhou M., Yu S., Jin Z., Zhao K. (2020). An overview of biodegradable nanomaterials and applications in vaccines. Vaccine.

[10] Zottig X., Côté-Cyr M., Arpin D., Archambault D., Bourgault S. (2020). Protein Supramolecular Structures: From Self-Assembly to Nanovaccine Design. Nanomaterials.

[11] Noad R.J., Roy P. (2003). Virus-like particles as immunogens. Trends Microbiol..

[12] Malik H., Khan F.H., Ahsan H. (2013). Human papillomavirus: Current status and issues of vaccination. Arch. Virol..

[13] Branco M.C., Sigano D.M., Schneider J.P. (2011). Materials from peptide assembly: Towards the treatment of cancer and transmittable disease. Curr. Opin. Chem. Biol..

[14] Eskandari S., Guerin T., Toth I., Stephenson R.J. (2017). Recent advances in self-assembled peptides: Implications for targeted drug delivery and vaccine engineering. Adv. Drug Deliv. Rev..

[15] Joo J., Poon C., Yoo S.P., Chung E.J. (2018). Shape Effects of Peptide Amphiphile Micelles for Targeting Monocytes. Molecules.

[16] Zaman M., Abdel-Aal A.-B.M., Fujita Y., Phillipps K.S.M., Batzloff M.R., Good M.F., Toth I. (2012). Immunological Evaluation of Lipopeptide Group A Streptococcus (GAS) Vaccine: Structure-Activity Relationship. PLoS ONE.

[17] Zaman M., Abdel-Aal A.-B.M., Fujita Y., Ziora Z.M., Batzloff M.R., Good M.F., Toth I. (2012). Structure–Activity Relationship for the Development of a Self-Adjuvanting Mucosally Active Lipopeptide Vaccine against Streptococcus pyogenes. J. Med. Chem..

[18] Riedel T., Ghasparian A., Moehle K., Rusert P., Trkola A., Robinson J.A. (2011). Synthetic Virus-Like Particles and Conformationally Constrained Peptidomimetics in Vaccine Design. ChemBioChem.

[19] Ghasparian A., Riedel T., Koomullil J., Moehle K., Gorba C., Svergun D.I., Perriman A.W., Mann S., Tamborrini M., Pluschke G. (2011). Engineered Synthetic Virus-Like Particles and Their Use in Vaccine Delivery. ChemBioChem.

[20] Tamborrini M., Geib N., Marrero-Nodarse A., Jud M., Hauser J., Aho C., Lamelas A., Zuniga A., Pluschke G., Ghasparian A. (2015). A Synthetic Virus-Like Particle Streptococcal Vaccine Candidate Using B-Cell Epitopes from the Proline-Rich Region of Pneumococcal Surface Protein A. Vaccines.

[21] Rudra J.S., Tian Y.F., Jung J.P., Collier J.H. (2010). A self-assembling peptide acting as an immune adjuvant. Proc. Natl. Acad. Sci. USA.

[22] Wen Y., Collier J.H. (2015). Supramolecular peptide vaccines: Tuning adaptive immunity. Curr. Opin. Immunol..

[23] Azmi F., Fuaad A.A., Giddam A.K., Batzloff M.R., Good M.F., Skwarczynski M., Toth I. (2014). Self-adjuvanting vaccine against group A streptococcus: Application of fibrillized peptide and immunostimulatory lipid as adjuvant. Bioorganic Med. Chem..

[24] Rudra J.S., Mishra J., Chong A.S., Mitchell R.A., Nardin E.H., Nussenzweig V., Collier J.H. (2012). Self-assembled peptide nanofibers raising durable antibody responses against a malaria epitope. Biomaterials.

[25] Babych M., Bertheau-Mailhot G., Zottig X., Dion J., Gauthier L., Archambault D., Bourgault S., Gauhier L. (2018). Engineering and evaluation of amyloid assemblies as a nanovaccine against the Chikungunya virus. Nanoscale.

[26] Kolpe A., Schepens B., Fiers W., Saelens X. (2017). M2-based influenza vaccines: Recent advances and clinical potential. Expert Rev. Vaccines.

[27] Watkins H.C., Rappazzo C.G., Higgins J.S., Sun X., Brock N., Chau A., Misra A., Cannizzo J.P., King M.R., Maines T.R. (2017). Safe Recombinant Outer Membrane Vesicles that Display M2e Elicit Heterologous Influenza Protection. Mol. Ther..

[28] Schepens B., De Vlieger D., Saelens X. (2018). Vaccine options for influenza: Thinking small. Curr. Opin. Immunol..

[29] De Filette M., Jou W.M., Birkett A., Lyons K., Schultz B., Tonkyro A., Resch S., Fiers W. (2005). Universal influenza A vaccine: Optimization of M2-based constructs. Virolology.

[30] Nguyen P.T., Zottig X., Sebastiao M., Bourgault S. (2017). Role of Site-Specific Asparagine Deamidation in Islet Amyloid Polypeptide Amyloidogenesis: Key Contributions of Residues 14 and 21. Biochemistry.

[31] De Carufel C.A., Quittot N., Nguyen P.T., Bourgault S. (2015). Delineating the Role of Helical Intermediates in Natively Unfolded Polypeptide Amyloid Assembly and Cytotoxicity. Angew. Chem..

[32] Zottig X., Al-Halifa S., Babych M., Quittot N., Archambault D., Bourgault S. (2019). Guiding the Morphology of Amyloid Assemblies by Electrostatic Capping: From Polymorphic Twisted Fibrils to Uniform Nanorods. Small.

[33] Hervé P.-L., Raliou M., Bourdieu C., Dubuquoy C., Petit-Camurdan A., Bertho N., Eleouet J.-F., Chevalier C., Riffault S. (2014). A Novel Subnucleocapsid Nanoplatform for Mucosal Vaccination against Influenza Virus That Targets the Ectodomain of Matrix Protein 2. J. Virol..

[34] Tovar J.D., Claussen R.C., Stupp S.I. (2005). Probing the Interior of Peptide Amphiphile Supramolecular Aggregates. J. Am. Chem. Soc..

[35] Bourgault S., Solomon J.P., Reixach N., Kelly J.W. (2011). Sulfated Glycosaminoglycans Accelerate Transthyretin Amyloidogenesis by Quaternary Structural Conversion. Biochemistry.

[36] Zhao R., So M., Maat H., Ray N.J., Arisaka F., Goto Y., Carver J.A., Hall D. (2016). Measurement of amyloid formation by turbidity assay—seeing through the cloud. Biophys. Rev..

[37] Sebastiao M., Quittot N., Bourgault S. (2017). Thioflavin T fluorescence to analyse amyloid formation kinetics: Measurement frequency as a factor explaining irreproducibility. Anal. Biochem..

[38] Wolfe L.S., Calabrese M.F., Nath A., Blaho D.V., Miranker A.D., Xiong Y. (2010). Protein-induced photophysical changes to the amyloid indicator dye thioflavin T. Proc. Natl. Acad. Sci. USA.

[39] Mao X.-B., Wang C.-X., Wu X.-K., Ma X.-J., Liu L., Zhang L., Niu L., Guo Y.-Y., Li D.-H., Yang Y.-L. (2011). Beta structure motifs of islet amyloid polypeptides identified through surface-mediated assemblies. Proc. Natl. Acad. Sci. USA.

[40] Batista F.D., Harwood N.E. (2010). Antigen presentation to B cells. F1000 Biol. Rep..

[41] Bennett K.M., Gorham R.D.J., Gusti V., Trinh L., Morikis D., Lo D.D. (2015). Hybrid flagellin as a T cell independent vaccine scaffold. BMC Biotechnol..

[42] Saar K.L., Mezzenga R. (2016). Amyloid Fibrils as Building Blocks for Natural and Artificial Functional Materials. Adv. Mater..

[43] Chiti F., Dobson C.M. (2017). Protein Misfolding, Amyloid Formation, and Human Disease: A Summary of Progress Over the Last Decade. Annu. Rev. Biochem..

[44] Bourgault S., Lim H.R., Buxbaum J.N., Kelly J.W., Price J.L., Reixach N. (2011). Mechanisms of transthyretin cardiomyocyte toxicity inhibition by resveratrol analogs. Biochem. Biophys. Res. Commun..

[45] Reixach N., Deechongkit S., Jiang X., Kelly J.W., Buxbaum J.N. (2004). Tissue damage in the amyloidoses: Transthyretin monomers and nonnative oligomers are the major cytotoxic species in tissue culture. Proc. Natl. Acad. Sci. USA.

[46] Otzen D.E. (2010). Functional amyloid. Prion.

[47] Godin E., Nguyen P.T., Zottig X., Bourgault S. (2019). Identification of a hinge residue controlling islet amyloid polypeptide self-assembly and cytotoxicity. J. Biol. Chem..

[48] Wen Y., Waltman A., Han H., Collier J.H. (2016). Switching the Immunogenicity of Peptide Assemblies Using Surface Properties. ACS Nano.

[49] Mora-Solano C., Wen Y., Han H., Chen J., Chong A.S., Miller M.L., Pompano R.R., Collier J.H. (2017). Active immunotherapy for TNF-mediated inflammation using self-assembled peptide nanofibers. Biomaterials.

[50] Jana M., Palencia C.A., Pahan K. (2008). Fibrillar amyloid-beta peptides activate microglia via TLR2: Implications for Alzheimer’s disease. J. Immunol..

[51] Westwell-Roper C., Denroche H.C., Ehses J.A., Verchere C.B. (2016). Differential Activation of Innate Immune Pathways by Distinct Islet Amyloid Polypeptide (IAPP) Aggregates. J. Boil. Chem..

[52] Reed S.G., Orr M.T., Fox C.B. (2013). Key roles of adjuvants in modern vaccines. Nat. Med..

[53] Eawate S., Babiuk L.A.B., Emutwiri G. (2013). Mechanisms of Action of Adjuvants. Front. Immunol..

[54] Kelley N., Jeltema D., Duan Y., He Y. (2019). The NLRP3 Inflammasome: An Overview of Mechanisms of Activation and Regulation. Int. J. Mol. Sci..

[55] Fernandes-Alnemri T., Kang S., Anderson C., Sagara J., Fitzgerald K.A., Alnemri E.S. (2013). Cutting edge: TLR signaling licenses IRAK1 for rapid activation of the NLRP3 inflammasome. J. Immunol..

[56] Nakanishi A., Kaneko N., Takeda H., Sawasaki T., Morikawa S., Zhou W., Kurata M., Yamamoto T., Akbar S.M.F., Zako T. (2018). Amyloid beta directly interacts with NLRP3 to initiate inflammasome activation: Identification of an intrinsic NLRP3 ligand in a cell-free system. Inflamm. Regen..

[57] Morikawa S., Kaneko N., Okumura C., Taguchi H., Kurata M., Yamamoto T., Osawa H., Nakanishi A., Zako T., Masumoto J. (2018). IAPP/amylin deposition, which is correlated with expressions of ASC and IL-1beta in beta-cells of Langerhans’ islets, directly initiates NLRP3 inflammasome activation. Int. J. Immunopathol. Pharmacol..

[58] Masters S.L., Dunne A., Subramanian S.L., Hull R.L., Tannahill G.M., Sharp F.A., Becker C., Franchi L., Yoshihara E., Chen Z. (2010). Activation of the NLRP3 inflammasome by islet amyloid polypeptide provides a mechanism for enhanced IL-1β in type 2 diabetes. Nat. Immunol..

[59] Stevens T.L., Bossie A., Sanders V.M., Fernandez-Botran R., Coffman R.L., Mosmann T.R., Vitetta E.S. (1988). Regulation of antibody isotype secretion by subsets of antigen-specific helper T cells. Nat. Cell Biol..

[60] Germann T., Bongartz M., Dlugonska H., Hess H., Schmitt E., Kolbe L., Kölsch E., Podlaski F.J., Gately M.K., Rüde E. (1995). Interleukin-12 profoundly up-regulates the synthesis of antigen-specific complement-fixing IgG2a, IgG2b and IgG3 antibody subclassesin vivo. Eur. J. Immunol..

[61] Lefeber D.J., Benaissa-Trouw B., Vliegenthart J.F.G., Kamerling J.P., Jansen W.T.M., Kraaijeveld K., Snippe H. (2003). Th1-Directing Adjuvants Increase the Immunogenicity of Oligosaccharide-Protein Conjugate Vaccines Related to Streptococcus pneumoniae Type 3. Infect. Immun..

[62] Gavin A.L., Hoebe K., Duong B., Ota T., Martin C., Beutler B., Nemazee D. (2006). Adjuvant-Enhanced Antibody Responses in the Absence of Toll-Like Receptor Signaling. Science.

[63] Denis J., Acosta-Ramirez E., Zhao Y., Hamelin M.-E., Koukavica I., Baz M., Abed Y., Savard C., Paré C., Lopez-Macias C.I.R. (2008). Development of a universal influenza A vaccine based on the M2e peptide fused to the papaya mosaic virus (PapMV) vaccine platform. Vaccine.

[64] Skwarczynski M., Toth I. (2014). Recent advances in peptide-based subunit nanovaccines. Nanomedicine.

